# Relationship of iodine excess with thyroid function in 6-year-old children living in an iodine-replete area

**DOI:** 10.3389/fendo.2023.1099824

**Published:** 2023-02-13

**Authors:** Yun Jeong Lee, Sun Wook Cho, Youn-Hee Lim, Bung-Nyun Kim, Johanna Inhyang Kim, Yun-Chul Hong, Young Joo Park, Choong Ho Shin, Young Ah Lee

**Affiliations:** ^1^Department of Pediatrics, Seoul National University Children’s Hospital, Seoul, Republic of Korea; ^2^Department of Pediatrics, Seoul National University College of Medicine, Seoul, Republic of Korea; ^3^Department of Internal Medicine, Seoul National University Hospital, Seoul, Republic of Korea; ^4^Department of Internal Medicine, Seoul National University Hospital College of Medicine, Seoul, Republic of Korea; ^5^Section of Environmental Health, Department of Public Health, University of Copenhagen, Copenhagen, Denmark; ^6^Institute of Environmental Medicine, Seoul National University Medical Research Center, Seoul, Republic of Korea; ^7^Environmental Health Center, Seoul National University College of Medicine, Seoul, Republic of Korea; ^8^Division of Children and Adolescent Psychiatry, Department of Psychiatry, Seoul National University Hospital, Seoul, Republic of Korea; ^9^Department of Psychiatry, Hanyang University Medical Center, Seoul, Republic of Korea; ^10^Department of Preventive Medicine, Seoul National University College of Medicine, Seoul, Republic of Korea

**Keywords:** iodine, thyroid hormone, urine, thyroid function test, child, Republic of Korea

## Abstract

**Background:**

Adequate iodine intake is essential for growing children, as both deficient and excessive iodine status can result in thyroid dysfunction. We investigated the iodine status and its association with thyroid function in 6-year-old children from South Korea.

**Methods:**

A total of 439 children aged 6 (231 boys and 208 girls) were investigated from the Environment and Development of Children cohort study. The thyroid function test included free thyroxine (FT4), total triiodothyronine (T3), and thyroid-stimulating hormone (TSH). Urine iodine status was evaluated using urine iodine concentration (UIC) in morning spot urine and categorized into iodine deficient (< 100 μg/L), adequate (100–199 μg/L), more than adequate (200–299 μg/L), mild excessive (300–999 μg/L), and severe excessive (≥ 1000 μg/L) groups. The estimated 24-hour urinary iodine excretion (24h-UIE) was also calculated.

**Results:**

The median TSH level was 2.3 μIU/mL, with subclinical hypothyroidism detected in 4.3% of patients without sex differences. The median UIC was 606.2 μg/L, with higher levels in boys (684 μg/L vs. 545 μg/L, *p* = 0.021) than girls. Iodine status was categorized as deficient (n = 19, 4.3%), adequate (n = 42, 9.6%), more than adequate (n = 54, 12.3%), mild excessive (n = 170, 38.7%), or severe excessive (n = 154, 35.1%). After adjusting for age, sex, birth weight, gestational age, body mass index z-score, and family history, both the mild and severe excess groups showed lower FT4 (β = − 0.04, *p* = 0.032 for mild excess; β = − 0.04, *p* = 0.042 for severe excess) and T3 levels (β = − 8.12, *p* = 0.009 for mild excess; β = − 9.08, *p* = 0.004 for severe excess) compared to the adequate group. Log-transformed estimated 24h-UIE showed a positive association with log-transformed TSH levels (β = 0.04, *p* = 0.046).

**Conclusion:**

Excess iodine was prevalent (73.8%) in 6-year-old Korean children. Excess iodine was associated with a decrease in FT4 or T3 levels and an increase in TSH levels. The longitudinal effects of iodine excess on later thyroid function and health outcomes require further investigation.

## 1 Introduction

Iodine is an essential micronutrient for the synthesis of thyroid hormone synthesis (1). Both iodine deficiency and excess can result in thyroid dysfunction, with a U-shaped association between iodine exposure and the risk of thyroid disease (2). Considering the critical role of thyroid function in childhood growth and development, children need an optimal iodine intake. The global health program has focused on intervention for iodine deficiency through salt iodization (3). However, with effective iodine fortification, concerns about iodine excess and its health impact have also been raised (4, 5). Iodine excess-induced hypothyroidism, resulting from failure to escape from the acute Wolff-Chaikoff effect in susceptible individuals has been reported (5) in addition to iodine-induced hyperthyroidism after iodine supplementation in iodine-deficient areas (6).

The association between iodine excess and thyroid dysfunction has been studied in the pediatric population (7–9), with some supporting results showing elevated thyroid stimulating hormone (TSH) levels in children exposed to iodine excess (8, 9). South Korea is an iodine-replete area (10) characterized by high seaweed consumption (11). According to the 2013–2015 Korean National Health and Nutrition Examination Survey, a high prevalence (64.9%) of iodine excess has been reported in Korean children and adolescents (8, 12).

In this study, we aimed to investigate the iodine status, using urine iodine measurements, and its association with thyroid function in 6-year-old Korean children.

## 2 Methods

### 2.1 Study population

This study was performed as a part of the Environment and Development of Children cohort study investigating the effects of early-life environmental risk factors on physical and neurobehavioral development (13). Among the 574 children who were examined at 6-years of age during 2015–2017 study period, 477 were evaluated for thyroid function and iodine status. Thirty-six children with multiple births and two diagnosed with thyroid disease (congenital hypothyroidism and Hashimoto’s thyroiditis, respectively) were excluded. Finally, 439 children were included in this study (**Supplementary Figure 1**). Informed consent was waived in accordance with the institutional review board of Seoul National University Hospital (IRB no. 1704-118-848).

### 2.2 Anthropometric assessments and questionnaires

Height, weight, and BMI z-scores were calculated using the 2007 Korean National Growth Charts, with overweight or obesity was defined as a body mass index (BMI) above the 85^th^ percentile (14). Data on personal and parental medical history, socioeconomic status (monthly household income and parental educational status), dietary supplement intake, and dairy product consumption were collected using structured questionnaires.

### 2.3 Evaluation of iodine status

Single spot urine samples were collected in the morning, after 8-hour of fasting, to measure the levels of UIC (μg/L) and urinary creatinine (Cr, mg/dL). UIC was measured by inductively coupled plasma mass spectrometry (ICP-MS), using a 7900 ICP-MS apparatus (Agilent Technologies, Santa Clara, CA, USA), and urinary Cr levels were measured by the kinetic alkaline picrate method (Architect TBA-C16000, Abbott, Abbott Park, IL, USA). The range of intra- and inter-assay coefficients of variation (CV) for UIC was 1.6-2.0% and 1.6-1.9%, respectively. The iodine status of the participants was assessed using the UIC (μg/L), iodine/Cr ratio (μg/g), and estimated 24-hour urine iodine excretion (24 h-UIE, μg/day). Children were categorized into four iodine status groups using the UIC, based on World Health Organization (WHO) recommendations: iodine deficiency (UIC < 100 μg/L), adequate (UIC 100–199 μg/L), more than adequate (UIC 200–299 μg/L), and excessive (UIC ≥ 300 μg/L) (3). The excessive group was further divided into the mild excessive (UIC 300–999 μg/L) and severe excessive (UIC ≥ 1000 μg/L) groups. The estimated 24 h-UIE (μg/day) was calculated from the iodine/Cr ratio using the following equation: estimated 24 h-UIE (μg/day) = iodine/Cr (μg/g) × predicted 24 h-Cr excretion (g/day). The predicted 24 h-Cr excretion (g/day) was determined using age- and anthropometry-based reference values (15).

### 2.4 Measurement of thyroid function

Thyroid function tests, including assays for free thyroxine (FT4), total triiodothyronine (T3), and TSH, were performed using a chemiluminescent microparticle immune assay on an ARCHITECT i2000 System (Abbott Korea, Seoul, Korea). The normal range was defined as 0.70–1.48 ng/dL (9.01–19.05 pmol/L) for FT4, 58–159 ng/dL (0.89–2.44 nmol/L) for T3, and 0.38–4.94 μIU/mL for TSH, respectively. The range of CVs for FT4, T3, and TSH was 2.0-2.6%, 3.0-4.4%, and 2.6-2.9%, respectively. Subclinical hypothyroidism was defined as TSH levels between 4.94–10 μIU/mL with normal FT4 levels.

### 2.5 Statistical analysis

All continuous variables were tested for normality and expressed as mean ± standard deviation or median with interquartile range (IQR). Iodine parameters and TSH levels were log-transformed for analysis. The participants’ characteristics were analyzed using the Student’s t-test or Mann–Whitney *U*-test for continuous variables, and the chi-squared test was used for categorical variables. Clinical characteristics and thyroid function were compared among the four iodine status groups (each group vs. the iodine-adequate group as a reference group), and a Bonferroni-corrected *p*-value of 0.017 was applied when performing multiple comparisons. The linear trend in thyroid function in the UIC categories was evaluated. We used generalized additive models (GAMs) to test the nonlinear relationship between iodine measurements and thyroid hormone levels. Linear and logistic regression analyses were used to evaluate the relationship between iodine status, thyroid hormone levels, and subclinical hypothyroidism. A directed acyclic graph (DAG) was used to visualize the causal relationships among variables and to identify potential confounding variables (**Supplementary Figure 2**) (16). The following variables were included in the multivariate model: age, sex, gestational age, birth weight, BMI z-scores, and parental history of thyroid disease. The R Statistical Software package (version 3.6.0; R Foundation for Statistical Computing, Vienna, Austria) was used for the statistical analyses, and the statistical significance was determined as *p* < 0.05.

## 3 Results

### 3.1 Clinical characteristics

**Table 1** shows the clinical characteristics of 439 children (231 boys) included in the analysis. The mean age was 5.9 ± 0.1 years and the mean BMI z-score was -0.1 ± 1.0. Twenty-one patients (4.8%) had a history of thyroid disease. Most parents (82.9% of mother and 85.2% of father) had high educational levels. The mean FT4 and T3 levels were 1.16 ± 0.11 ng/dL and 148.1 ± 18.5 ng/dL, respectively. The median TSH level was 2.29 μIU/mL (range, 0.53–8.59 μIU/mL). None of the patients showed overt hypothyroidism and 19 (4.3%) had subclinical hypothyroidism. No sex-related differences in thyroid function were observed. All children with subclinical hypothyroidism had a normal stature, with three children (15.8%) being overweight and two (10.5%) obese. None of these children showed clinical signs of hypothyroidism.

**Table 1:** Clinical characteristics of study participants.

Variables	Total (N = 439)	Boys (n = 231)	Girls (n = 208)
Age, years	5.9 ± 0.1	5.9 ± 0.1	5.9 ± 0.1
Gestational age, weeks	39.3 ± 1.3	39.3 ± 1.3	39.3 ± 1.3
Birth weight, kg	3.3 ± 0.4	3.3 ± 0.4^*^	3.2 ± 0.4^*^
Height, cm	115.9 ± 4.5	116.5 ± 4.6^*^	115.2 ± 4.2^*^
Weight, kg	21.4 ± 3.3	21.6 ± 3.2	21.1 ± 3.3
Body mass index, kg/m^2^	15.9 ± 1.8	15.9 ± 1.7	15.9 ± 2.0
Body mass index z-score	−0.06 ± 1.03	−0.14 ± 0.99	0.02 ± 1.07
Parental history of thyroid disease, n (%)	21 (4.8)	13 (5.6)	8 (3.9)
Monthly household income (> 4,000K KRW), n (%)	303 (69.0)	151 (65.4)	152 (73.1)
Paternal education level ≥ college, n (%)	374 (85.2)	197 (85.3)	177 (85.1)
Maternal education level ≥ college, n (%)	364 (82.9)	191 (82.7)	173 (83.2)
FT4, ng/dL	1.16 ± 0.11	1.17 ± 0.11	1.16 ± 0.11
T3, ng/dL	148.1 ± 18.5	147.6 ± 18.9	148.7 ± 18.0
TSH, μIU/mL	2.29 (1.71–2.98)	2.29 (1.63–2.98)	2.30 (1.76–2.99)
Subclinical hypothyroidism, n (%)	19 (4.3)	11 (4.8)	8 (3.9)
UIC, μg/L	606.2 (292.4–1446.4)	683.6 (317.1–1570.8) ^*^	554.8 (236.8–1272.7) ^*^
Iodine status group^a^, n (%)	19/42/54/324 (4.3/9.6/12.3/73.8)	6/17/29/179 (2.6/7.4/12.6/77.5)	13/25/25/145 (6.3/12.0/12.0/69.7)
Iodine/Cr ratio, μg/g	826.1 (427.6–1856.1)	825.3 (434.2–2079.7)	838.2 (417.8–1690.8)
Estimated 24 h-UIE, μg/day	313.4 (155.9–646.3)	333.4 (166.2–802.4)	284.4 (145.4–590.8)

Data are expressed as the mean ± standard deviation, median (interquartile range), or number (%).

^*^p < 0.05 between boys and girls.

a Iodine status groups were as follows: iodine deficient (UIC < 100 μg/L), adequate (UIC 100 to 199 μg/L), more than adequate (UIC 200 to 299 μg/L), and excessive (UIC ≥ 300 μg/L).

UIC, urine iodine concentration; Cr, creatinine; 24h-UIE, 24-hour urinary iodine excretion; FT4, free thyroxine; T3, total triiodothyronine; TSH, thyroid stimulating hormone.

### 3.2 Comparison among iodine status categories

The median UIC was 606.2 μg/L (IQR, 292.4–1446.4 μg/L), with a between-sex difference (683.6 μg/L in boys vs. 554.8 μg/L in girls, *p* = 0.021, **Table 1**). The distribution of iodine status categories was as follows: iodine deficient (n = 19, 4.3%), adequate (n = 42, 9.6%), more than adequate (n = 54, 12.3%), or excessive (n = 324, 73.8%). The median iodine/Cr and estimated 24 h-UIE levels were 826.1 μg/g and 313.4 μg/day, respectively, with no between-sex difference. There were no differences in clinical characteristics among the four iodine categories (**Supplementary Table 1**). In addition, dietary supplement intake or proportion of high dairy product consumption (≥ 2 cups/day) did not differ among the four groups.

As shown in **Table 2**, we compared the three iodine status categories (iodine deficient, more than adequate, and excessive groups) with the reference category (iodine adequate group). The iodine excessive group showed lower T3 levels (147.2 vs. 155.1 mg/dL, *p* = 0.010), as revealed by the Bonferroni correction (*p* = 0.017). Interestingly, 17 (89.5%) of the 19 children with subclinical hypothyroidism had excessive iodine. However, FT4 and log-transformed TSH (log-TSH) levels and the risk of subclinical hypothyroidism did not differ among the four iodine categories.

**Table 2:** Thyroid function status according to iodine status.

	Deficient (UIC < 100 μg/L)	Adequate (UIC 100 to 199 μg/L)	More than adequate (UIC 200 to 299 μg/L)	Excessive (UIC ≥ 300 μg/L)	*P* for trend	*P* for between group comparison^a^ (each group vs. adequate group as reference)		
						*P*a	*P*b	*P*c
Total, n	19	42	54	324		–	–	–
FT4, ng/dL	1.16 ± 0.09	1.20 ± 0.10	1.18 ± 0.12	1.16 ± 0.11	0.125	0.147	0.412	0.032
T3, ng/dL	150.7 ± 17.0	155.1 ± 20.4	147.2 ± 18.1	147.2 ± 18.2	0.034	0.414	0.048	0.010
Log-transformed TSH, μIU/mL	0.72 ± 0.39	0.87 ± 0.37	0.67 ± 0.47	0.84 ± 0.45	0.213	0.155	0.028	0.740
Subclinical hypothyroidism, n (%)	0 (0.0)	1 (2.4)	1 (1.9)	17 (5.3)	0.127	0.999	0.999	0.668

*P*a, adequate vs. deficient; *P*b, adequate vs. more than adequate; *P*c, adequate vs. excessive.

a Bonferroni correction was applied for multiple comparisons and the significance level was set at p < 0.017.

Data are expressed as the mean (standard deviation) or number (%).

UIC, urine iodine concentration, FT4, free thyroxine; T3, total triiodothyronine; TSH, thyroid stimulating hormone.

### 3.3 Relationship between iodine status and thyroid function

**Figure 1:**
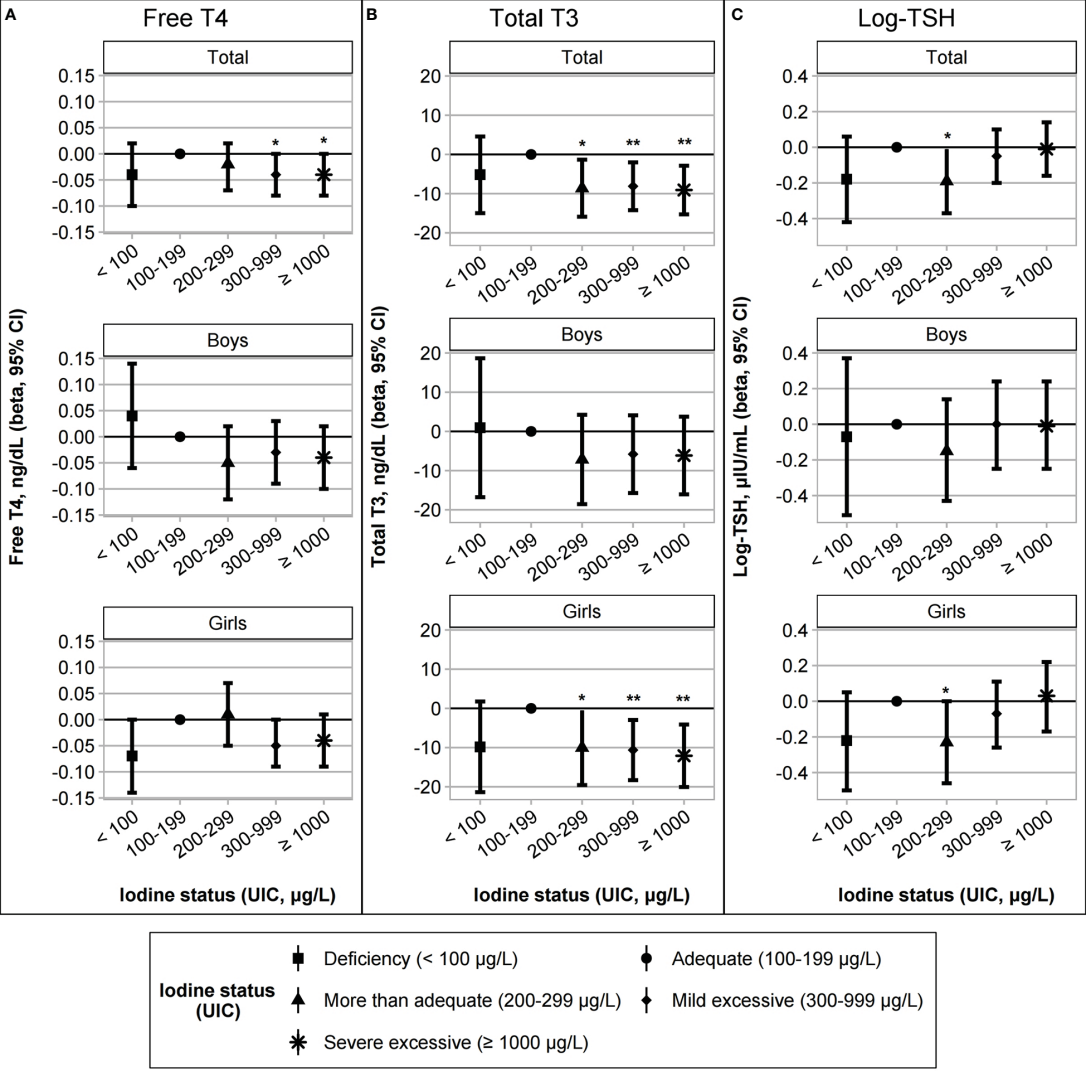
Association between iodine status and thyroid function **(A)** free T4, **(B)** total T3, and **(C)** log-transformed TSH levels. The iodine status groups were categorized as deficient (UIC < 100 μg/L, n = 19), adequate (UIC 100–199 μg/L, n = 42), more than adequate (UIC 200–299 μg/L, n = 54), mild excessive (UIC 300–999 μg/L, n = 170), or severe excessive (UIC ≥ 1000 μg/L, n = 154). Regression models for the total group were adjusted for age, sex, gestational age, birth weight, body mass index z-scores, and a parental history of thyroid disease. The sex-stratified models were adjusted for age, gestational age, birth weight, body mass index z-scores, and parental history of thyroid disease. ^*^p < 0.05; ^**^p < 0.01.

Due to the high prevalence of iodine excess, iodine status was classified into five categories [deficient, adequate, more than adequate, mild excessive (UIC of 300–999 μg/L, n = 170, 38.7%), and severe excessive (UIC ≥ 1000 μg/L, n = 154, 35.1%)]. The results of the univariate analysis are described in **Supplementary Tables 2, 3**. After adjusting for age, sex, gestational age, birth weight, BMI z-scores, and parental history of thyroid disease, a multivariate-adjusted model was constructed to compare thyroid function among the five iodine categories. Compared to the reference category (iodine adequate group), the more than adequate group showed lower T3 levels (β = –8.62, *p* = 0.021, **Figure 1B**) and log-TSH levels (β = –0.19, *p* = 0.035, **Figure 1C**), and both mild and severe excessive groups showed lower FT4 (β = –0.04, *p* = 0.032; mild excessive group: β = –0.04, *p* = 0.042 for the severe excessive group; **Figure 1A**) and T3 levels (β = –8.12, *p* = 0.009, for the mild excessive group; β = –9.08, *p* = 0.004 for the severe excessive group; **Figure 1B**). In a sex-stratified analysis, these associations for iodine excess were significant in girls but not in boys (**Figure 1**; **Table 3**). In girls, FT4 and T3 levels were lower in the iodine-deficient group than in the iodine-adequate group with marginal significance. Due to the small number of children with adequate and more than adequate group, we performed a sensitivity analysis by combining the two groups as a reference category. The results showed the similar trends of lower FT4 and T3 and higher TSH levels among the iodine excess group compared to reference group (**Supplementary Table 4**).

**Table 3:** Association between the iodine status and thyroid hormone levels after adjusting for covariates (multivariate models).

Category	UIC, μg/L	N	FT4, ng/dL (β, 95% CI)			T3, ng/dL (β, 95% CI)			Log-transformed TSH, μIU/mL (β, 95% CI)		
			Total (n = 439)	Boys (n = 231)	Girls (n = 208)	Total (n = 439)	Boys (n = 231)	Girls (n = 208)	Total (n = 439)	Boys (n = 231)	Girls (n = 208)
Deficient	<100	19	–0.04 (–0.10, 0.02)	0.04 (–0.06, 0.14)	–0.07 (–0.14, 0.00)	–5.21 (–14.98, 4.57)	0.94 (–16.78, 18.65)	–9.81 (–21.37, 1.75)	–0.18 (–0.42, 0.06)	–0.07 (–0.51, 0.37)	–0.22 (–0.50, 0.05)
Adequate	100–199	42	0 [Ref]			0 [Ref]			0 [Ref]		
More than adequate	200–299	54	–0.02 (–0.07, 0.02)	–0.05 (–0.12, 0.02)	0.01 (–0.05, 0.07)	–8.62 (–15.89, –1.34)^*^	–7.16 (–18.56, 4.24)	–10.02 (–19.55, –0.49)^*^	–0.19 (–0.37,–0.01)^*^	–0.15 (–0.43, 0.14)	–0.23 (–0.46, 0.00)^*^
Mild excessive	300–999	170	–0.04 (–0.08, 0.00)^*^	–0.03 (–0.09, 0.03)	–0.05 (–0.09, 0.00)	–8.12 (–14.22, –2.03)^**^	–5.81 (–15.72, 4.10)	–10.63 (–18.30, –2.96)^**^	–0.05 (–0.20, 0.10)	0.00 (–0.25, 0.24)	–0.07 (–0.26, 0.11)
Severe excessive	≥ 1000	154	–0.04 (–0.08, 0.00)^*^	–0.04 (–0.10, 0.02)	–0.04 (–0.09, 0.01)	–9.08 (–15.29, –2.88)^**^	–6.15 (–16.05, 3.75)	–12.09 (–20.06, –4.12)^**^	–0.01 (–0.16, 0.14)	–0.01 (–0.25, 0.24)	0.03 (–0.17, 0.22)

UIC, urine iodine concentration, FT4, free thyroxine; T3, total triiodothyronine; TSH, thyroid stimulating hormone; N, number; CI, confidence interval; Ref, Reference.

Regression models for the total group were adjusted for age, sex, gestational age, birth weight, body mass index z–scores, and parental history of thyroid disease. Sex–stratified models were adjusted for age, gestational age, birth weight, body mass index z–scores, and parental history of thyroid disease.

^*^p < 0.05; ^**^p < 0.01.

We investigated the nonlinear relationship between iodine measurements and thyroid hormone levels using GAM but did not reveal clear nonlinear relationships (**Supplementary Figure 3**). To focus on the association between iodine excess and thyroid function, we analyzed iodine-sufficient or-excessive children (n = 420) after excluding iodine-deficient children (n = 19). Results of the univariate analysis are reported in **Supplementary Table 5**, showing positive associations between continuous iodine variables and log-TSH levels in girls. After adjusting for covariates, log-transformed iodine/Cr (log-iodine/Cr) and log-transformed estimated 24 h-UIE (log-estimated 24 h-UIE) levels were significantly associated with log-TSH levels (β = 0.05, *p* = 0.035 for log-iodine/Cr; β = 0.04, *p* = 0.046 for log-estimated 24 h-UIE, **Table 4**). In a sex-stratified analysis, a positive relationship between log-iodine/Cr and log-estimated 24 h-UIE and log-TSH levels was found only in girls (*p* < 0.05, **Table 4**) but not in boys. FT4 and T3 levels were not associated with log-transformed UIC, iodine/Cr, nor estimated 24 h-UIE levels in either sex. The risk of subclinical hypothyroidism was not associated with continuous iodine variables (**Supplementary Table 6**).

**Table 4:** Association between continuous iodine variables and thyroid hormone levels after adjusting for covariates.

Variables	FT4, ng/dL (β, 95% CI)			T3, ng/dL (β, 95% CI)			Log-transformed TSH, μIU/mL (β, 95% CI)		
	Total (n = 420)	Boys (n = 225)	Girls (n = 195)	Total (n = 420)	Boys (n = 225)	Girls (n = 195)	Total (n = 420)	Boys (n = 225)	Girls (n = 195)
**Log-transformed UIC, μg/L**	−0.01 (−0.02, 0.00)	−0.01 (−0.02, 0.01)	0.00 (−0.02, 0.01)	−1.49 (−3.18, 0.19)	−0.60 (−2.99, 1.79)	−2.38 (−4.79, 0.04)	0.03 (−0.01, 0.07)	0.01 (−0.05, 0.07)	0.05 (0.00, 0.11)
**Log-transformed iodine/Cr, μg/g**	−0.01 (−0.02, 0.01)	−0.01 (−0.02, 0.01)	−0.01 (−0.02, 0.01)	−1.23 (−2.98, 0.52)	−0.57 (−3.01, 1.88)	−1.85 (−4.46, 0.76)	0.05 (0.00, 0.09)^*^	0.03 (−0.03, 0.09)	0.08 (0.02, 0.14)^**^
**Log-transformed estimated 24 h-UIE, μg/day**	−0.01 (−0.02, 0.00)	−0.01 (−0.02, 0.01)	−0.01 (−0.02, 0.01)	−1.23 (−2.98, 0.51)	−0.56 (−2.98, 1.85)	−1.89 (−4.51, 0.74)	0.04 (0.00, 0.09)^*^	0.03 (−0.04, 0.09)	0.08 (0.02, 0.14)^*^

UIC, urine iodine concentration; Cr, creatinine; 24h-UIE, 24-hour urinary iodine excretion; FT4, free thyroxine; T3, total triiodothyronine; TSH, thyroid stimulating hormone.

The iodine-deficient group (n = 19) was excluded from analysis. Regression models for the total group were adjusted for age, sex, gestational age, birth weight, body mass index z-scores, and parental history of thyroid disease. The sex-stratified models were adjusted for age, gestational age, birth weight, body mass index z-scores, and parental history of thyroid disease.

^*^p < 0.05; ^**^p < 0.01.

## 4 Discussion

Among 6-year-old children living in South Korea, iodine excess was prevalent (73.8%), with a median UIC level of 606 μg/L. Only 9.6% of the children showed adequate iodine status, with a low frequency (4.3%) of iodine deficiency. Compared to the iodine adequate group, the iodine excessive group exhibited lower FT4 and T3 levels, and the log-estimated 24 h-UIE showed a positive association with log-TSH levels. In a sex-stratified analysis, this trend was only significant in girls. Although iodine excess was reported to be prevalent in Korean children, 6 to 19 years of age, this is the first study showing a decreasing trend of FT4 and T3 levels within the normal range in the iodine excess group and an increasing trend of log-TSH levels, with higher urine iodine excretion in Korean prepubertal children.

The high levels of the median UIC (606 μg/L (IQR, 292.4–1466.4]) and the high prevalence of iodine excess (73.8%) in 6-year-old children were comparable to previous pediatric reports in children 6 to 19 years of age (median UIC of 449 μg/L and 64.9% of iodine excess) (8). This was higher than the UIC of 274 ug/L in the adult population, according to the same database from the Korean National Health and Nutrition Examination Survey from 2013–2015 (17). Excessive iodine status has been reported in Korean children, ranging from 54.9% to 76.7% prevalence of iodine excess and 328 to 458 μg/L median UIC (18–20). Common dietary sources of iodine in Korea include seaweeds, such as sea mustard, laver, kelp, as well as pickled vegetables, milk, and dairy products (11). However, there were no significant differences in dairy product consumption among iodine status groups in this study. Higher UIC levels in boys than in girls may result from higher consumption of iodine-rich food and higher energy intake in boys (21). Considering the absence of sex differences in Cr-adjusted iodine variables, sex differences in UIC may be due to differences in urine volume or hydration status (12, 17).

The decreasing trend in FT4 and T3 levels with increasing TSH levels in the iodine excess group suggests a possible risk for iodine excess-associated hypothyroidism. Although the prevalence of subclinical hypothyroidism was not significantly different among the iodine groups (2.4% in the adequate and 5.3% in the excess group), 89.5% (17 of 19 children) of children with subclinical hypothyroidism had an iodine excess. Several pediatric studies have supported the effect of iodine excess on a shift in FT4 or T3 to lower levels (8), TSH towards higher levels (8, 9, 22), and an increased risk of subclinical hypothyroidism (23). However, some studies have reported no significant associations (7, 24). The prevalence of subclinical hypothyroidism among iodine groups also varied according to the geographic region and age group. A previous nationwide Korean study showed significantly different frequencies of subclinical hypothyroidism: 3.9% in the iodine adequate and 6.6% in the excess group (8). A Chinese study also reported similar results, with a higher prevalence of subclinical hypothyroidism in high iodine area (6.7%, median UIC of 1030 μg/L) than in adequate iodine area (0.7%, median UIC of 123 μg/L) (23). However, an international study performed in 12 countries showed similar rates of subclinical hypothyroidism in the iodine adequate (0.5%) and excess (0.6%) group (9). A meta-analysis found that chronic exposure to excess iodine increased the odds ratio for subclinical and overt hypothyroidism in adults but not children, possibly due to the high heterogeneity of the included pediatric studies (4). Previous pediatric studies were limited by reporting the crude differences in thyroid hormone levels among UIC groups (8, 9, 22, 24). Only a few pediatric studies have performed the adjusted relationship between iodine status and thyroid function (7, 23). The use of multivariate-adjusted regression models strengthened the findings in our study.

We found lower FT4 levels in girls in the iodine-deficient group than in the adequate group. However, our results were limited by the small number of participants in iodine-deficient groups. Iodine deficiency is associated with hypothyroidism and goiter in children (25). Thus, both iodine deficiency and excess can lead to impaired thyroid hormone production, suggesting a U-shaped correlation (2). A recent nationwide study in Korean children and adolescents (10–19 years of age) also showed a U-shape and an inverted U-shape correlation between serum TSH and FT4 levels and UIC, respectively (8).

The mechanisms underlying iodine-induced thyroid dysfunction include failure to escape Wolff-Chaikoff effects or iodine-induced thyroid autoimmunity (5), although there is a possibility of additional mechanisms. Acute iodine excess can cause a transient decrease in thyroid hormone production by inhibiting thyroid peroxidase activity or intrathyroidal deiodinase activity, called the Wolff-Chaikoff effect (5). Healthy individuals can maintain normal thyroid function by escaping the Wolff-Chaikoff effect within several days, through a decrease in sodium iodine symporter expression. However, failure of this adaptation can lead to iodine-induced hypothyroidism in susceptible populations, such as infants, the elderly, or those with thyroid diseases (5, 26, 27). In addition, high iodine levels may induce thyroid autoimmunity, leading to elevated TSH levels and hypothyroidism (26). As this study included healthy children, iodine-induced thyroid dysfunction was not definite in our study population. However, a decreasing trend in thyroid hormone levels was observed in the iodine-excessive group.

Only girls showed a significant association between iodine status and thyroid function in this study. A Korean adult study reported that the correlation between iodine status and the thyroid disease was only significant in women, suggesting that women may be more susceptible to excessive iodine exposure (28). Estrogen, directly and indirectly, affects thyroid gland proliferation and function, including iodine uptake through the sodium-iodine symporter and thyroid peroxidase activity (29). However, because this study included prepubertal children, the mechanisms underlying sex differences remain unclear.

In this study, a higher BMI z-score and lower gestational age were associated with higher serum T3 levels in our sample of 6-year-old children. With regard to childhood obesity, the positive relationship between BMI and T3 levels can be explained by an adaptation process to increase resting energy expenditure or elevated deiodinase activity (30–32). The relationship between prematurity and childhood thyroid function has previously been reported, showing trends towards decrease in free T4 and an increase in T3 and TSH levels (33, 34), although the underlying mechanisms remains to be determined.

We used UIC, iodine/Cr ratio, and the estimated 24h-UIE as iodine variables in this study. Although measurement of 24h-UIE is the most reliable method to evaluate iodine status, it is difficult to apply in field studies (35, 36). UIC from spot urine has been the most widely used biomarker for iodine status in populations as >90% of dietary iodine is excreted by urine, although UIC is influenced by urine volume and hydration status (35). UIC related to urinary Cr has been used to overcome this limitation to determine iodine status. In children, anthropometry-based 24-h urinary Cr reference values can be used to estimate the 24h-UIE from spot urine (15). Several pediatric studies have reported that the estimated 24h-UIE better reflects measured 24h-UIE than UIC, providing a valid and reliable alternative to measured 24h-UIE (37, 38). In this study, we used the estimated 24h-UIE to complement UIC and identified a significant positive association between the estimated 24h-UIE and TSH levels.

The limitations of our study need to be acknowledged in the interpretation of findings to practice. Foremost is the cross-sectional design and relatively small sample size, including the small proportion of participants in the non-iodine excess group, which did not allow us to evaluate the relationship between iodine deficiency and thyroid function. Further studies using a prospective design and focusing on environmental sources of iodine excess are needed to generalize the adverse effects of iodine excess on health outcomes and to suggest preventive strategies for iodine excess. Second, the single-spot urine collection used in this study to assess UIC for iodine status could not reflect within-day and day-to-day variations in urine iodine excretion. Although we could not collect 24 h urine samples or repeatedly collect spot urine samples, an ideal way to provide a valid estimate of iodine status (39), spot urine samples collected in the morning after overnight fasting can eliminate within-day variations. In addition, we obtained similar results regardless of which iodine variables (UIC or urinary Cr-adjusted iodine status) used to examine the association between iodine status and thyroid function (35). Third, we could not evaluate thyroid autoantibodies in all study populations. However, thyroid autoantibodies were not detected in our children with subclinical hypothyroidism. Considering the low prevalence of positive thyroid autoantibodies (2.3%) in Korean children and adolescents (8), the modifying effect of thyroid autoimmunity is insignificant in our 6-year-old children. Fourth, potential confounders, such as exposure to endocrine disrupting chemicals (EDCs) or heavy metals that can affect thyroid dysfunction, were not considered in this study. The mixed effects of various thyroid disrupting chemicals, including both iodine and other EDCs, need further investigation.

In conclusion, iodine was deficient in 4.3%, adequate in 21.9%, and excessive in 73.8% of 6-year-old children living in South Korea during 2015–2017. Furthermore, excess iodine was associated with decreased FT4 or T3 levels and increased TSH levels, particularly in girls. The long-term health effects of iodine excess remain to be determined, considering the high prevalence of iodine excess in Korean children. Further studies to determine the optimal iodine intake are required.

## Data availability statement

The datasets presented in this article are not readily available because the fundamental results of the study are reported in the paper and supporting information. The raw data cannot be shared publicly due to ethical restrictions because they contain potentially sensitive information. The data are available upon reasonable request from the Scientific Research Committee. Requests to access the datasets should be directed to YJL, yjlee103@naver.com.

## Ethics statement

The studies involving human participants were reviewed and approved by Institutional Review Board of Seoul National University Hospital (IRB no. 1704-118-848). Written informed consent from the participants’ legal guardian/next of kin was not required to participate in this study in accordance with the national legislation and the institutional requirements.

## Author contributions

YJL carried out the initial analyses, drafted the initial manuscript, and reviewed and revised the manuscript. SWC, Y-HL, B-NK, JIK, Y-CH, YJP, and CHS conceptualized and designed the study, coordinated and supervised data collection, and critically reviewed the manuscript for important intellectual content. YAL conceptualized and designed the study, collected data, and reviewed and revised the manuscript. All authors contributed to the article and approved the submitted version.
