# Supplementary material for: Relationship of iodine excess with thyroid function in 6-year-old children living in an iodine-replete area
Source: Front Endocrinol (Lausanne). 2023 Feb 13;14:1099824. doi: 10.3389/fendo.2023.1099824 (PMC9968830; doi:10.3389/fendo.2023.1099824)
Supplement: Supplementary file 1 [file DataSheet_1.docx]

Supplementary Material

1. **Supplementary Figures and Tables**
   1. **Supplementary Tables**

**Supplementary Table 1. Clinical characteristics of participants according to iodine status**

|  | Deficiency  (n = 19) | Adequate  (n = 42) | More than adequate  (n = 54) | Excess  (n = 324) |
| --- | --- | --- | --- | --- |
| UIC range, μg/L | < 100 | 100–199 | 200–299 | 300–999 |
| Boys, n (%) | 6 (31.6) | 17 (40.5) | 29 (53.7) | 179 (55.2) |
| Gestational age, weeks | 39.5 ± 1.1 | 39.3 ± 1.4 | 39.1 ± 1.5 | 39.3 ± 1.3 |
| Birth weight, kg | 3.4 ± 0.4 | 3.3 ± 0.4 | 3.3 ± 0.4 | 3.3 ± 0.4 |
| Height, cm | 116.1 ± 4.5 | 115.7 ± 3.8 | 116.2 ± 4.5 | 115.9 ± 4.6 |
| Weight, kg | 21.2 ± 2.2 | 20.9 ± 2.3 | 21.3 ± 3.2 | 21.4 ± 3.5 |
| Body mass index, kg/m^2^ | 15.7 ± 1.3 | 15.6 ± 1.4 | 15.7 ± 1.7 | 15.9 ± 1.9 |
| Body mass index z-score | -0.04 ± 0.83 | -0.15 ± 0.94 | -0.12 ± 0.93 | -0.04 ± 1.07 |
| Overweight and/or obesity, n (%) | 1 (5.3) | 0 (0.0) | 4 (7.4) | 15 (4.6) |
| Parental history of thyroid disease, n (%) | 0 (0.0) | 1 (2.4) | 2 (3.7) | 18 (5.6) |
| Monthly household income (> 4,000 K KRW), n (%) | 13 (68.4) | 28 (66.7) | 37 (68.5) | 225 (69.4) |
| Paternal education level ≥ college, n (%) | 17 (89.5) | 35 (83.3) | 47 (87.0) | 275 (84.9) |
| Maternal education level ≥ college, n (%) | 18 (94.7) | 35 (83.3) | 48 (88.9) | 263 (81.2) |
| Dietary supplement intake, n (%) | 2 (10.5) | 8 (19.0) | 9 (16.7) | 74 (22.8) |
| High consumption of dairy product (milk or yogurt ≥ 2 cups/day), n (%) | 5 (26.3) | 22 (52.4) | 23 (42.6) | 146 (45.2) |

Data are expressed as mean ± standard deviation, median (interquartile range), or number (%). UIC, urinary iodine concentration

**Supplementary Table 2. Association between iodine status and thyroid hormone level (univariate models)**

| Category | UIC, μg/L | No. | FT4, ng/dL (β, 95% CI) | | | T3, ng/dL (β, 95% CI) | | | Log-transformed TSH, μIU/mL  (β, 95% CI) | | |
| --- | --- | --- | --- | --- | --- | --- | --- | --- | --- | --- | --- |
|  |  |  | Total  (n = 439) | Boys  (n = 231) | Girls  (n = 208) | Total  (n = 439) | Boys  (n = 231) | Girls  (n = 208) | Total  (n = 439) | Boys  (n = 231) | Girls  (n = 208) |
| Adequate | 100–199 | 42 | 0 [Reference] | | | 0 [Reference] | | | 0 [Reference] | | |
| Deficient | <100 | 19 | –0.04 (–0.10, 0.02) | 0.04 (–0.06, 0.14) | –0.08 (–0.15, 0.00)^*^ | –4.42 (–14.39, 5.54) | 0.46 (–17.16, 18.07) | –7.02 (–19.06, 5.02) | –0.15 (–0.39, 0.09) | –0.11 (–0.55, 0.33) | –0.17 (–0.45, 0.10) |
| More than adequate | 200–299 | 54 | –0.02 (–0.06, 0.02) | –0.05 (–0.12, 0.02) | 0.01 (–0.04, 0.07) | –7.89 (–15.31, –0.48)^*^ | –7.98 (–19.31, 3.35) | –7.03 (–16.99, 2.93) | –0.20 (–0.37, –0.02)^*^ | –0.15 (–0.43, 0.13) | –0.23 (–0.46, –0.01)^*^ |
| Mild excessive | 300–999 | 170 | –0.04 (–0.07, 0.00)^*^ | –0.03 (–0.09, 0.03) | –0.05 (–0.09, 0.00) | –8.04 (–14.26, –1.83)^*^ | –6.47 (–16.29, 3.34) | –9.11 (–17.16, –1.05)^*^ | –0.05 (–0.20, 0.10) | –0.02 (–0.26, 0.22) | –0.06 (–0.25, 0.12) |
| Severe excessive | ≥ 1000 | 154 | –0.04 (–0.08, 0.00)^*^ | –0.04 (–0.10, 0.01) | –0.04 (–0.08, 0.01) | –7.71 (–13.99, –1.44)^*^ | –6.23 (–16.04, 3.58) | –8.65 (–16.95, –0.34)^*^ | 0.00 (–0.15, 0.15) | –0.01 (–0.26, 0.23) | 0.05 (–0.14, 0.24) |

UIC, urine iodine concentration, FT4, free thyroxine; T3, total triiodothyronine; TSH, thyroid stimulating hormone

*^*^p* < 0.05 by univariate regression analysis with the reference category (iodine adequate group)

**Supplementary Table 3. Association of covariates with thyroid hormone levels (univariate model)**

|  | FT4, ng/dL (β, 95% CI) | T3, ng/dL (β, 95% CI) | Log-transformed TSH, μIU/mL (β, 95% CI) |
| --- | --- | --- | --- |
| Age, years | 0.00 (–0.01, 0.00) | –1.04 (–2.10, 0.02) | –0.02 (–0.04, 0.01) |
| Boys (vs. girls) | 0.01 (–0.01, 0.03) | –1.09 (–4.55, 2.37) | –0.05 (–0.13, 0.04) |
| Gestational age, weeks | –0.01 (–0.01, 0.00) | –1.37 (–2.71, –0.04)^*^ | 0.02 (–0.01, 0.06) |
| Birth weight, kg | –0.01 (–0.04, 0.01) | –0.5 (–4.64, 3.64) | 0.06 (–0.04, 0.16) |
| Body mass index z–score | 0.00 (–0.01, 0.01) | 3.49 (1.84, 5.15)^**^ | 0.04 (0.00, 0.08) |
| Parental history of thyroid disease | 0.00 (–0.05, 0.05) | 1.74 (–6.36, 9.84) | 0.00 (–0.20, 0.19) |

*^*^p* < 0.05; *^**^p* < 0.01

**Supplementary Table 4. Association of iodine status with thyroid hormone levels after adjusting for covariates (adequate-more than adequate group as reference)**

| Category | UIC, μg/L | No | FT4, ng/dL (β, 95% CI) | | | T3, ng/dL (β, 95% CI) | | | Log-transformed TSH, μIU/mL  (β, 95% CI) | | |
| --- | --- | --- | --- | --- | --- | --- | --- | --- | --- | --- | --- |
|  |  |  | Total  (n = 439) | Boys  (n = 231) | Girls  (n = 208) | Total  (n = 439) | Boys  (n = 231) | Girls  (n = 208) | Total  (n = 439) | Boys  (n = 231) | Girls  (n = 208) |
| Deficient | <100 | 19 | -0.03 (-0.08, 0.03) | 0.07 (-0.02, 0.17) | -0.07 (-0.14, -0.01)^*^ | -0.41 (-9.35, 8.53) | 5.40 (-10.85, 21.65) | -4.92 (-15.58, 5.75) | -0.07 (-0.29, 0.15) | 0.02 (-0.38, 0.42) | -0.06 (-0.31, 0.19) |
| Adequate-more than adequate | 100-299 | 96 | 0 [Reference] |  |  | 0 [Reference] |  |  | 0 [Reference] |  |  |
| Mild excessive | 300-999 | 170 | -0.03 (-0.06, 0.00)^*^ | 0.00 (-0.04, 0.04) | -0.05 (-0.09, -0.01)^*^ | -3.26 (-7.80, 1.27) | -1.30 (-8.14, 5.53) | -5.73 (-11.87, 0.41) | 0.06 (-0.05, 0.17) | 0.09 (-0.08, 0.26) | 0.05 (-0.09, 0.20) |
| Severe excessive | ≥ 1000 | 154 | -0.03 (-0.05, 0.00) | -0.01 (-0.05, 0.03) | -0.04 (-0.08, 0.00) | -4.21 (-8.87, 0.45) | -1.65 (-8.48, 5.18) | -7.12 (-13.58, -0.65)^*^ | 0.10 (-0.01, 0.21) | 0.08 (-0.09, 0.25) | 0.17 (0.01, 0.32)^*^ |

UIC, urine iodine concentration, FT4, free thyroxine; T3, total triiodothyronine; TSH, thyroid stimulating hormone

Regression models for total group were adjusted for age, sex, gestational age, birth weight, body mass index z-scores, and parental history of thyroid disease. Sex-stratified models were adjusted for age, gestational age, birth weight, body mass index z-scores, and parental history of thyroid disease.

*^*^p* < 0.05

**Supplementary Table 5. Association between continuous iodine variables and thyroid hormone levels (univariate model)**

| Variables | FT4, ng/dL (β, 95% CI) | | | T3, ng/dL (β, 95% CI) | | | Log-transformed TSH, μIU/mL (β, 95% CI) | | |
| --- | --- | --- | --- | --- | --- | --- | --- | --- | --- |
|  | Total  (n = 420) | Boys  (n = 225) | Girls  (n = 195) | Total  (n = 420) | Boys  (n = 225) | Girls  (n = 195) | Total  (n = 420) | Boys  (n = 225) | Girls  (n = 195) |
| Log-transformed UIC, μg/L | –0.01 (–0.02, 0.00) | –0.01 (–0.02, 0.01) | –0.01 (–0.02, 0.01) | –1.16 (–2.85, 0.54) | –0.40 (–2.73, 1.94) | –2.01 (–4.52, 0.49) | 0.03 (–0.01, 0.07) | 0.01 (–0.04, 0.07) | 0.06 (0.00, 0.12)^*^ |
| Log-transformed iodine/Cr, μg/g | –0.01 (–0.02, 0.01) | –0.01 (–0.02, 0.01) | –0.01 (–0.02, 0.01) | –0.83 (–2.61, 0.95) | –0.26 (–2.64, 2.12) | –1.61 (–4.30, 1.08) | 0.05 (0.01, 0.09)^*^ | 0.03 (–0.03, 0.09) | 0.09 (0.03, 0.15)^**^ |
| Log-transformed estimated 24 h-UIE, μg/day | –0.01 (–0.02, 0.00) | –0.01 (–0.02, 0.01) | –0.01 (–0.03, 0.01) | –0.79 (–2.55, 0.97) | –0.26 (–2.60, 2.07) | –1.48 (–4.18, 1.22) | 0.05 (0.01, 0.09)^*^ | 0.02 (–0.04, 0.08) | 0.09 (0.03, 0.15)^**^ |

UIC, urine iodine concentration; Cr, creatinine; 24 h–UIE, 24–hour urinary iodine excretion; FT4, free thyroxine; T3, total triiodothyronine; TSH, thyroid stimulating hormone

Iodine–deficient group (n = 19) was excluded for the analysis.

*^*^p* < 0.05; *^**^p* < 0.01

**Supplementary Table 6. Association between continuous iodine variables and subclinical hypothyroidism**

| Variables | Odds ratio for subclinical hypothyroidism (95% CI) | | | | | |
| --- | --- | --- | --- | --- | --- | --- |
|  | Total (n = 420) | | Boys (n = 225) | | Girls (n = 195) | |
|  | Univariate | Multivariate^a^ | Univariate | Multivariate^a^ | Univariate | Multivariate^a^ |
| Log-transformed UIC, μg/L | 1.37 (0.89, 2.10) | 1.29 (0.83, 2.00) | 1.71 (0.98, 2.99) | 1.70 (0.96, 3.03) | 0.96 (0.48, 1.94) | 0.94 (0.45, 1.95) |
| Log-transformed iodine/Cr, μg/g | 1.30 (0.83, 2.04) | 1.48 (0.82, 2.67) | 1.55 (0.88, 2.74) | 1.48 (0.82, 2.67) | 0.97 (0.46, 2.05) | 0.97 (0.43, 2.17) |
| Log-transformed estimated 24 h-UIE, μg/day | 1.27 (0.82, 1.98) | 1.42 (0.79, 2.56) | 1.47 (0.84, 2.58) | 1.42 (0.79, 2.56) | 0.97 (0.46, 2.06) | 0.94 (0.42, 2.11) |

UIC, urine iodine concentration; Cr, creatinine; 24 h–UIE, 24–hour urinary iodine excretion

^a^Multivariate models for total group were adjusted for age, sex, gestational age, birth weight, body mass index z–scores, and parental history of thyroid disease. Sex–stratified models were adjusted for age, gestational age, birth weight, body mass index z–scores, and parental history of thyroid disease

- 1. **Supplementary Figures**

**Supplementary Figure 1. Flowchart of the participants**

**Supplementary Figure 2.** **Directed acyclic graphs showing the relationship between iodine status and thyroid function.** Directed acyclic graphs (DAGs) show the hypothesized causal relationship between iodine status (yellow circles), thyroid function (blue circles), and covariates (white and pink circles). The proposed adjustment variables are indicated by white circles.

**Supplementary Figure 3. Association of continuous iodine measurements with thyroid hormone levels: (A) free T4, (B) total T3, and (C) log-transformed TSH levels (univariate models).** A generalized additive model (GAM) was used to obtain a smoothing curve. The shaded areas represent the 95% CIs.
