# Supplementary figures and images for: Relationship of iodine excess with thyroid function in 6-year-old children living in an iodine-replete area
Source: Front Endocrinol (Lausanne). 2023 Feb 13;14:1099824. doi: 10.3389/fendo.2023.1099824 (PMC9968830; doi:10.3389/fendo.2023.1099824)

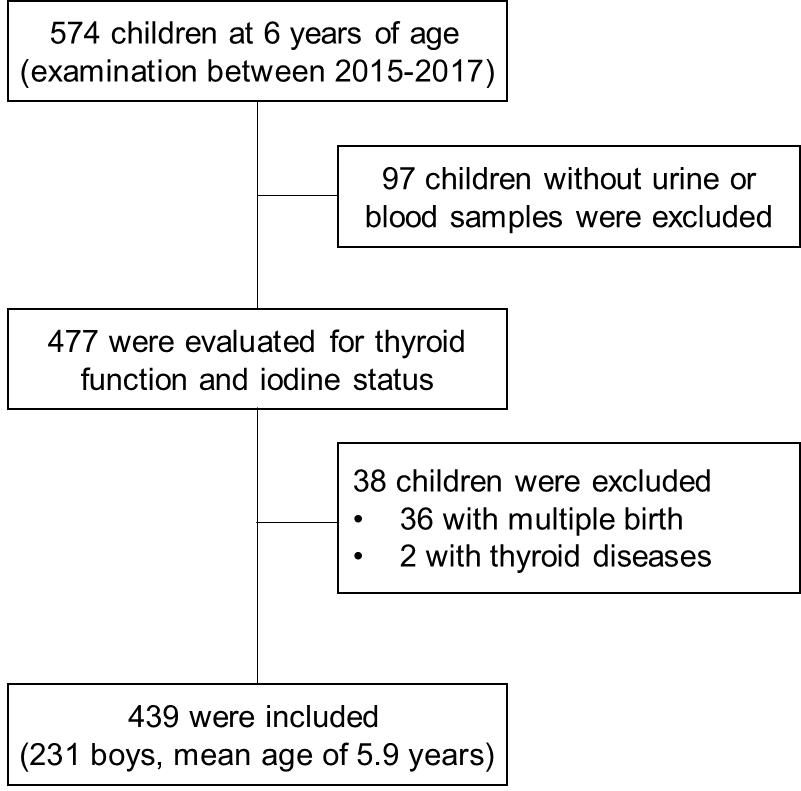

Supplement: Supplementary file 2 [file Image_1.tif]

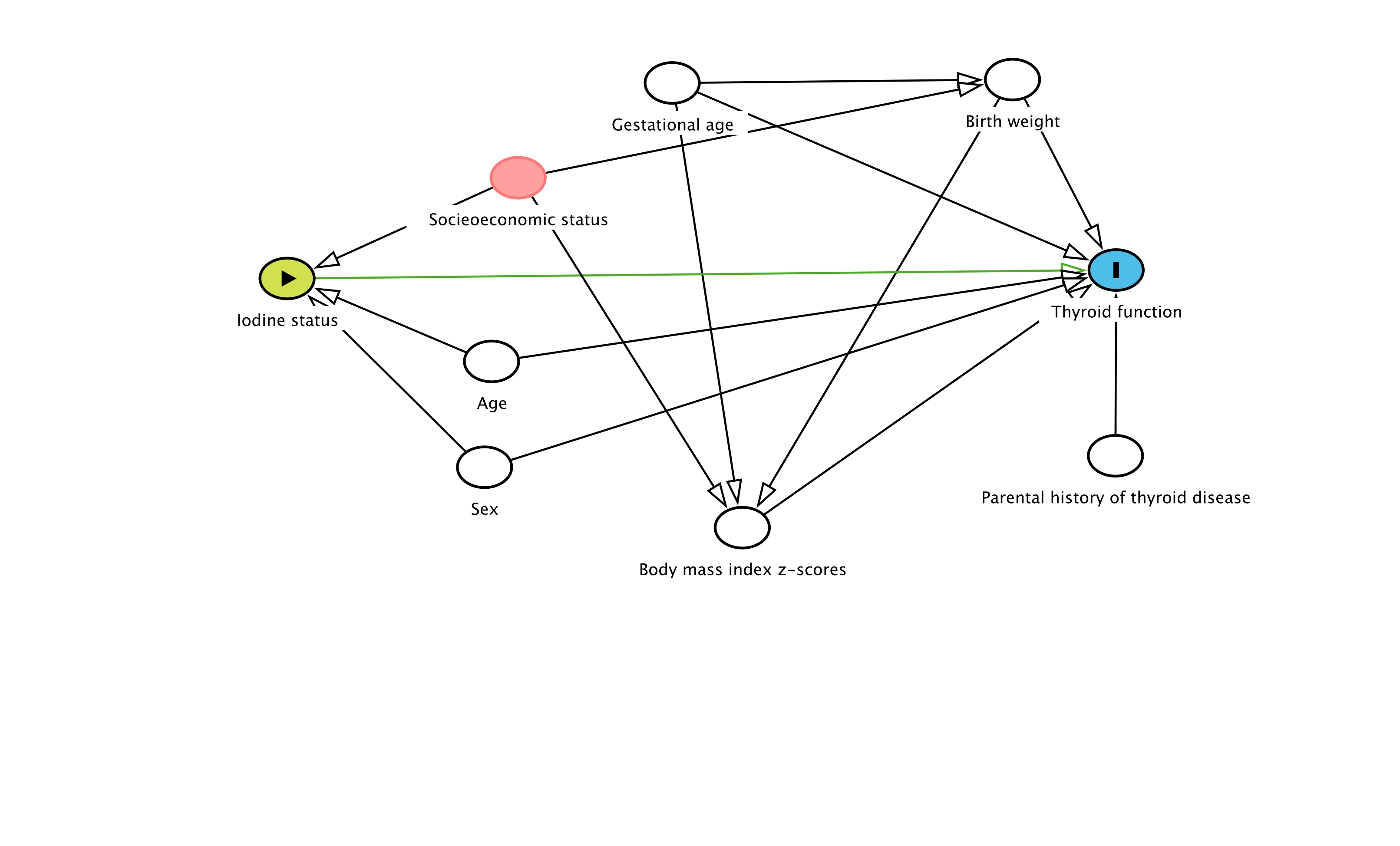

Supplement: Supplementary file 3 [file Image_2.tiff]

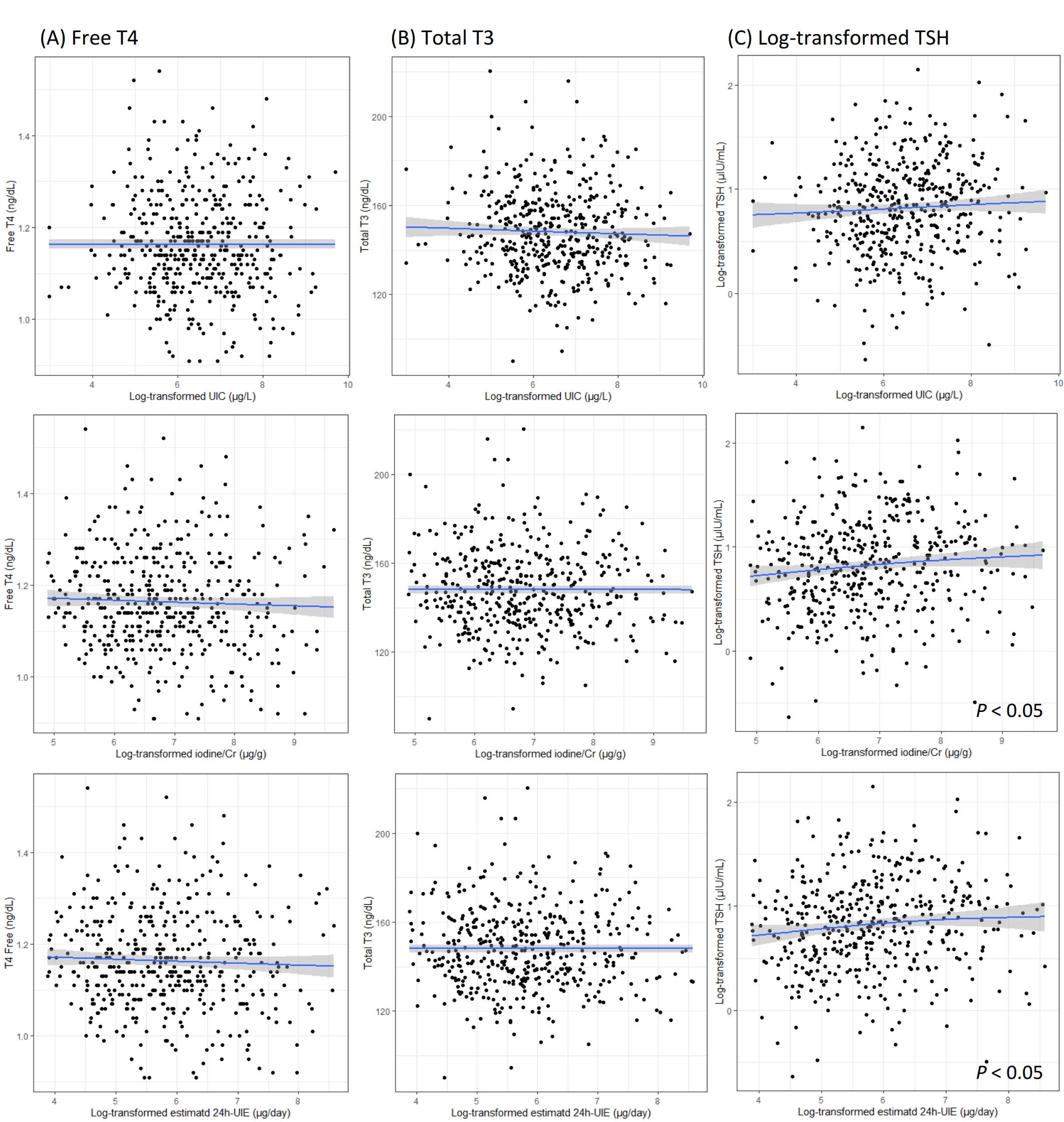

Supplement: Supplementary file 4 [file Image_3.tiff]
